# Trans-scleral fixation of Cionni - modified capsule tension ring

**DOI:** 10.4103/0301-4738.67049

**Published:** 2010

**Authors:** Ashok Sharma

**Affiliations:** Cornea Center, SCO 833-834 Sector 22A, Chandigarh-160 022, India

Dear Editor,

I read with great interest the article entitled, “Results of intraocular lens implantation with capsular tension ring in subluxated crystalline or cataractous lenses in children,” by Das *et al*.[1] I congratulate the authors for an excellent study on a very relevant issue. The authors have shown 20/40 or better vision in 16 of 18 (89%) pediatric eyes, having a zonular dialysis of 90 to 210 degrees with Polymethyl methacrylate (PMMA) or an acrylic intraocular lens (IOL), implanted with a capsular tension ring (CTR). However, I believe that patients with zonular dialysis > 180 degrees might have shown better results if Cionni-modified CTR with a single or double eyelet was inserted and sutured to the sclera. Trans-scleral fixation of Cionni-modified CTR and IOL complex may provide better fixation, centration, and long-term stability in these cases with zonular dialysis > 180 degrees. The procedure will, in addition, eliminate the possible necessity of a second procedure of scleral fixation of one haptic, to correct the increased subluxation and IOL decentration in the postoperative period.[2]
